# Supplementary material for: cDNA Cloning of Feline PIWIL1 and Evaluation of Expression in the Testis of the Domestic Cat
Source: Int J Mol Sci. 2023 May 26;24(11):9346. doi: 10.3390/ijms24119346 (PMC10253428; doi:10.3390/ijms24119346)
Supplement: Supplementary file 1 [file ijms-24-09346-s001.zip › ijms-1956738-supplementary.pdf]

International Journal of
Molecular Sciences

MDPI

Supplementary Tables

Table S1: PIWIL1 Isoform Alignment Between Species

a. PIWIL1-2

ORGANISM	Amino Acids (total)	Total Alignment[a] (%)	PIWI Domain Alignment[a] (%)	PAZ domain Alignment[a] (%)
Homo sapiens	861	95.349	97.072	96.610
Mus musculus	862	96.984	98.423	97.458
Canis lupus familiaris	861	98.258	99.550	98.305
Sus scrofa	861	98.142	99.550	97.458

b. PIWIL1-1

ORGANISM	Amino Acids (total)	Total Alignment[a] (%)	PIWI Domain Alignment[a] (%)	PAZ domain Alignment[a] (%)
Homo sapiens	861	79.356	74.194	96.610
Mus musculus	862	80.842	75.346	97.458
Canis lupus familiaris	861	82.040	76.498	98.305
Sus Scrofa	861	81.929	76.498	97.458

[a]Table compiled using MUSCLE alignment with 8 allowable iterations. Compared sequences were gathered from the NCBI database, and GenBank accession numbers are as follows: H. sapiens (NP_004755), M. musculus (NP_067286.1), C. familiarus (ALT31692.1) and S. scrofa (NP_001181902.1).

Table S1. Comparison of identity (%) and similarity (%) of the 861-amino acid sequence of *F. catus* PIWIL1-isoform 2 (PIWIL1-2: **a**) and PIWIL1-1: **b**) with known PIWIL1 proteins from other mammalian species.

High levels of sequence homology between *Felis catus* PIWIL1-2 and PIWIL1 from other mammals examined implies strong evolutionary conservation, suggesting that the PIWIL1-2 variant is most likely to represent the original feline PIWIL1, as it is found in multiple mammalian species. Striking sequence differences in the PIWI domain of PIWIL1-1 when compared both within species (to PIWIL1-2) and with other mammalian PIWIL1, suggests that this isoform may play an alternate role in PIWI biology when expressed in the feline testis.

Table S2: Summary of Primers Utilized

FPL1: Feline PIWIL1, FPL2: Feline PIWIL2

Primer name	Purpose	Vector	Sequence (5'-3')
FPL1-iso1-fwd	Cloning	pcDNA3.1	GATCGGTACCATGACCGGGCGAG (Acc651)
FPL1-iso1-rvse	Cloning	pcDNA3.1	GATCGGATCCCTAAGAAACGGGT (BamH1)
FPL1-iso2-fwd	Cloning	pcDNA3.1	GATCGGTACCATGACCGGGCGAG (Acc651)
FPL1-iso2-rvse	Cloning	pcDNA3.1	GATCGGATCCTCAGAGGTAGTACAGG (BamH1)
FPL2-fwd	Cloning	pcDNA3.1	GATCGGTACCATGGATCCTGTCCG (Acc651)
FPL2-Rvse	Cloning	pcDNA3.1	GATCCTCGAGTCACAGGAAGAACAGG (Xho1)
FelPIWI1S1-F	Sequencing	N/A	ACATCACCAACTTGTTTGCAGT
FelPIWI1S2-F	Sequencing	N/A	CGCGAAGTGGGAAGACTCAT
FelPIWI1S3-F	Sequencing	N/A	GGCAAGCAGCAGACAGTCAT
RPS5fwd	qPCR	N/A	TCACTGGTGAGAACCCCCT
RPS5rvse	qPCR	N/A	CCTGATTCACACGGCGTAG
fSDHAfwd	qPCR	N/A	GCAGAACCTGATGCTTTGTG
fSDHArvse	qPCR	N.A	GGAAAGGGTGTGCTTCCTCC
GAPDHfwd	qPCR	N/A	GCTGAACGGGAAGCTCACT
GAPDHrvse	qPCR	N/A	CGAAGGTGGAAGAGTGGGTG
qFPL1-iso1-fwd	qPCR	N/A	AAAAACCTCCCTGACTTGCTCTGA
qFPL1-iso1-rvse	qPCR	N/A	CCAGCAGTTGGTATCGTTGCA
qFPL1-iso2-fwd	qPCR	N/A	ACGAGTGAACGCCCGATTTT
qFPL1-iso2-rvse	qPCR	N/A	ACAGCTTGTACGTCAGCCTC

Table S3: Summary of Antibodies Utilized

Antibody	Company and catalogue #	LOT#	RRID#	Use
PIWIL1	Abcam #12337	GR220659-1 GR185575-1	AB_470241	WB, IHC
PIWIL1	Abcam #105393	GR41071-9	AB_10861173	WB, IP
Actin	Cell Signaling #4967S	04-2015	AB_330288	WB
IgG	Millipore #12-370	2458935	AB_145841	IP

Table S4: Age and Breed Characteristics of Sample Sources

Sample Number	Age (Maturity)	Breed
36	11 months (Mature)	Domestic Short Hair (DSH)
51	3 months (Immature)	DSH
54	3 months (Immature)	DSH
55	3 months (Immature)	Unknown
60	1 year (Mature)	DSH
61	1 year (Mature)	DSH
77	4 years (Mature)	DSH

78	3 years (Mature)	DSH
83	4 years (Mature)	DSH
121	2 months (Immature)	DSH
130	2 months (Immature)	DSH
131	3 months (Immature)	DSH
